# Ultrasound-guided gluteal nerves electrical stimulation to enhance strength and power in individuals with chronic knee pain: a randomized controlled pilot trial

**DOI:** 10.3389/fmed.2024.1410495

**Published:** 2024-07-03

**Authors:** Francesco Sartori, Pedro Luiz Flores Fagnani, Laia Monne-Guasch, Giovanni La Cagnina, Javier Picañol, Albert Puig-Diví

**Affiliations:** ^1^Blanquerna School of Health Sciences, Ramon Llull University, Barcelona, Spain; ^2^Independent Researcher, Barcelona, Spain; ^3^Ecole Polytechnique Fédérale de Lausanne, Lausanne, Switzerland; ^4^Laboratory of Neurophysiology, Biomedicine Department, Faculty of Medicine, Institute of Neurosciences, University of Barcelona, Barcelona, Spain; ^5^Department of Health Sciences, Tecnocampus, Pompeu Fabra University, Mataró, Spain

**Keywords:** ultrasound-guided percutaneous peripheral nerve stimulation, chronic pain, knee pain, neuromodulation, TENS, rehabilitation medicine, electrophysiology, electrical stimulation

## Abstract

**Introduction:**

Various pathophysiological contexts can be accompanied by weakness, arthrogenic muscle inhibition, and even disability. In this scenario, peripheral nerve stimulation has been studied not only for pain management but also for the improvement of neuromuscular parameters. For this purpose, the use of Transcutaneous Electrical Nerve Stimulation (TENS) has typically been investigated, but recently, the use of ultrasound-guided percutaneous peripheral nerve stimulation (pPNS) has gained popularity. In this regard, electrical stimulation has a predisposition to activate Type II muscle fibers and has been shown to be capable of generating short-term potentiation by increasing calcium sensitivity. However, the evidence of pPNS applied in humans investigating such variables is rather limited.

**Objectives:**

This pilot study aimed to assess the feasibility of the methodology and explore the potential of pPNS in enhancing hip extension performance in individuals suffering from knee pain, comparing it with TENS.

**Methods:**

Twelve participants were divided into pPNS and TENS groups, undergoing pre- and post-intervention assessments of peak concentric power (W), strength (N), execution speed (m/s), and one-repetition maximum (1RM) (kg) estimation. For pPNS, two needles were positioned adjacent to the superior and inferior gluteal nerves under ultrasound guidance. For TENS, electrodes were positioned between the posterosuperior iliac spine and the ischial tuberosity, and halfway between the posterosuperior iliac spine and the greater trochanter. The interventions consisted of 10 stimulations of 10 s at a frequency of 10 Hz with a pulse width of 240 μs, with rest intervals of 10 s between stimulations.

**Results:**

Peripheral nerve stimulation significantly improved concentric power at 30% (*p* = 0.03) and 50% (*p* = 0.03) of 1RM, surpassing TENS, which showed minimal changes. No significant strength differences were observed post-intervention in either group.

**Conclusion:**

This work presents evidence where pPNS applied to the gluteal nerves results in an enhanced performance of hip extension at submaximal loads. However, this improvement does not seem to be reflected in short-term changes in the estimation of the 1RM by the force-velocity profile.

## Introduction

A spectrum of injuries can be accompanied by arthrogenic muscle inhibition (AMI), which is identified by a compromised ability for muscle activation within a specific area (1). This phenomenon is linked to several processes, including joint swelling, inflammation, pain, and structural damage, among others (1). In this regard, the majority of evidence pertaining to this concept is primarily associated with knee injuries and the extensor musculature of this joint. For instance, anterior cruciate ligament injury, among others, has been linked to quadriceps inhibition (1, 2). From a neurophysiological perspective, it is proposed that this mechanism may be underpinned by alterations in spinal reflexes or changes at the supraspinal level (1). Specifically, findings include modifications in the gamma loop function, the flexion reflex (which may play a role via reciprocal inhibition), non-reciprocal inhibition mediated by Group Ib afferents, enhanced motor cortex excitability, descending pathways of facilitation and inhibition, and the motivational states relating to maximal voluntary effort. Moreover, pain has been shown to induce controversial neuromuscular adaptations (3). In this context, nociceptive information can modulate sensory afferents, thereby affecting the gamma loop (4), increasing the flexor reflex hyperexcitability (5), and stimulating Ib interneurons, which may facilitate the autogenic inhibition of the involved motoneurons (6).

The evidence pertaining to AMI is primarily delineated in the context of the knee, yet occurrences within the gluteal musculature have also been observed (7). However, there exists conflicting evidence and ongoing debate regarding gluteal weakness in various clinical contexts. On the one hand, several studies indicate that individuals experiencing pain may exhibit increased gluteal activity (8–11). On the other hand, gluteal weakness and inhibition have been observed in various pathologies, including knee pain (12). Indeed, deficits in isometric strength and rate of force development (RFD) in hip abduction and extension have been identified in patients with patellofemoral syndrome (13). Furthermore, patients with knee pain exhibit a 50% reduction in gluteal activation during dynamic tasks such as jumping (14). Simultaneously, assessing hip strength may aid in identifying athletes at risk of injury (15) and the engagement of gluteal musculature in certain phases of dynamic tasks such as sprinting may reduce the risk of hamstring strains (16). Over the last decade, this debate has led to the popularization of interventions targeting the gluteal musculature, aimed at treating and/or preventing several clinical scenarios (17–19).

Various approaches based on peripheral stimulation have been explored to ameliorate AMI. For instance, traditional transcutaneous electrical nerve stimulation (TENS) has been shown to enhance excitability within the quadriceps motor neuron pool in healthy subjects and to increase the quadriceps central activation ratio in individuals with osteoarthritis (20–22). In a broader sens, TENS as a therapy aimed at reducing inhibition may aid in improving muscle activation, potentially benefiting certain clinical situations (23, 24). However, in recent years, the application of percutaneous peripheral nerve stimulation (pPNS) has emerged as a method to improve pain management and functional outcomes (25, 26). This technique involves the ultrasound guided insertion of a needle to electrically stimulate targeted peripheral nerves. Unlike TENS, pPNS can stimulate axons more specifically. The mechanisms of action for pPNS remain a subject of ongoing investigation, but basic research has revealed effects on both the peripheral and central nervous systems (27). Initially, the principal mechanism of action was hypothesized to align with Melzack and Wall’s gate control theory, wherein the stimulation of large-diameter, low-threshold Ab non-nociceptive fibers leads to the activation of interneurons in the dorsal horn of the spinal cord, modulating nociceptive information from A gamma and C fibers (28, 29). Beyond this, research has identified central mechanisms whereby pPNS influences GABAergic, glycinergic, and serotonergic signaling (30, 31), induces endogenous inhibition via dorsal wide dynamic range neurons (32), suppresses glutamatergic AMPA receptor activity in certain animal models (33), and reduces spinothalamic tract activity following repeated stimulation (34). At the peripheral level, pPNS modifies nociceptive afferent activity, induces silent periods in induced neuroma models (35), locally regulates neurotransmitters, endorphins, and inflammatory mediators, and even decreases ectopic afferent activity (29).

Regarding its use for muscle recruitment, there are non-exclusive models suggesting that peripheral stimulation could be beneficial by its ability to induce contractile activity within the same motor unit (MU) pool, eliciting supramaximal temporal recruitment depending on the stimulation frequency, and facilitating synchronous recruitment of adjacent fibers (36). This method serves as a complement to voluntary effort due to its unconventional spatial recruitment of the involved motor units (36). Similar to TENS, it could decrease the activity of inhibitory Ib interneurons or enhance contraction capabilities through pain reduction (20, 37). However, the exploration of pPNS in this specific context remains limited and the research emerging in recent years indicates that it may be beneficial for muscle recruitment, strength, and performance enhancement. For instance, when applied to the femoral nerve in conjunction with an exercise program, it has been shown to increase vertical jump height (38). Alvarez-Prats et al. (39) reported improvements in quadriceps strength following pPNS. Similarly, Requena et al. (40) found that pPNS acutely enhances the isokinetic torque and power of knee extensors. In a related vein, de la Cruz-Torres et al. observed that the ultrasound-guided application of low-frequency current to the muscle belly of the first flexor muscle led to strength increases in balance and resistance tests among dancers (41). Additional evidence concluded that pPNS applied to acupuncture points adjacent to the peroneal nerve resulted in ankle dorsiflexion strength gains (42). In more complex conditions such as multiple sclerosis, pPNS has been suggested to improve grip strength (43). However, the body of evidence remains relatively sparse, and the methodological limitations observed in the designs of the cited studies must be taken into account.

Given the ongoing debate regarding the potential use of pPNS for enhancing strength and performance, this study investigates whether its application to the gluteal nerves could improve gluteal function in patients with chronic knee pain compared to the traditional use of transcutaneous electrical nerve stimulation. This will clarify whether there is a need for more in-depth research into an emerging cost-effective methodology capable of enhancing the function of the gluteal musculature in individuals experiencing pain.

## Materials and methods

### Methodology

This study adhered to the extended Consolidated Standards of Reporting Trials (CONSORT) guidelines for randomized pilot studies and feasibility trials (44) and followed the Standard Protocol Items: Recommendations for Interventional Trials (SPIRIT) guidelines (45). It received prior approval from the Blanquerna School of Health Sciences Research Ethics Committee (CER) (Barcelona, Spain) under the reference N° 20230901. The study was carried in alignment with the principles outlined in the Declaration of Helsinki, ensuring ethical conduct and participant safety. All participants provided written informed consent before being enrolled in the study. Additionally, this study was officially registered on ClinicalTrials.gov, bearing the identification number NCT06340035.

### Design

**Figure 1:**
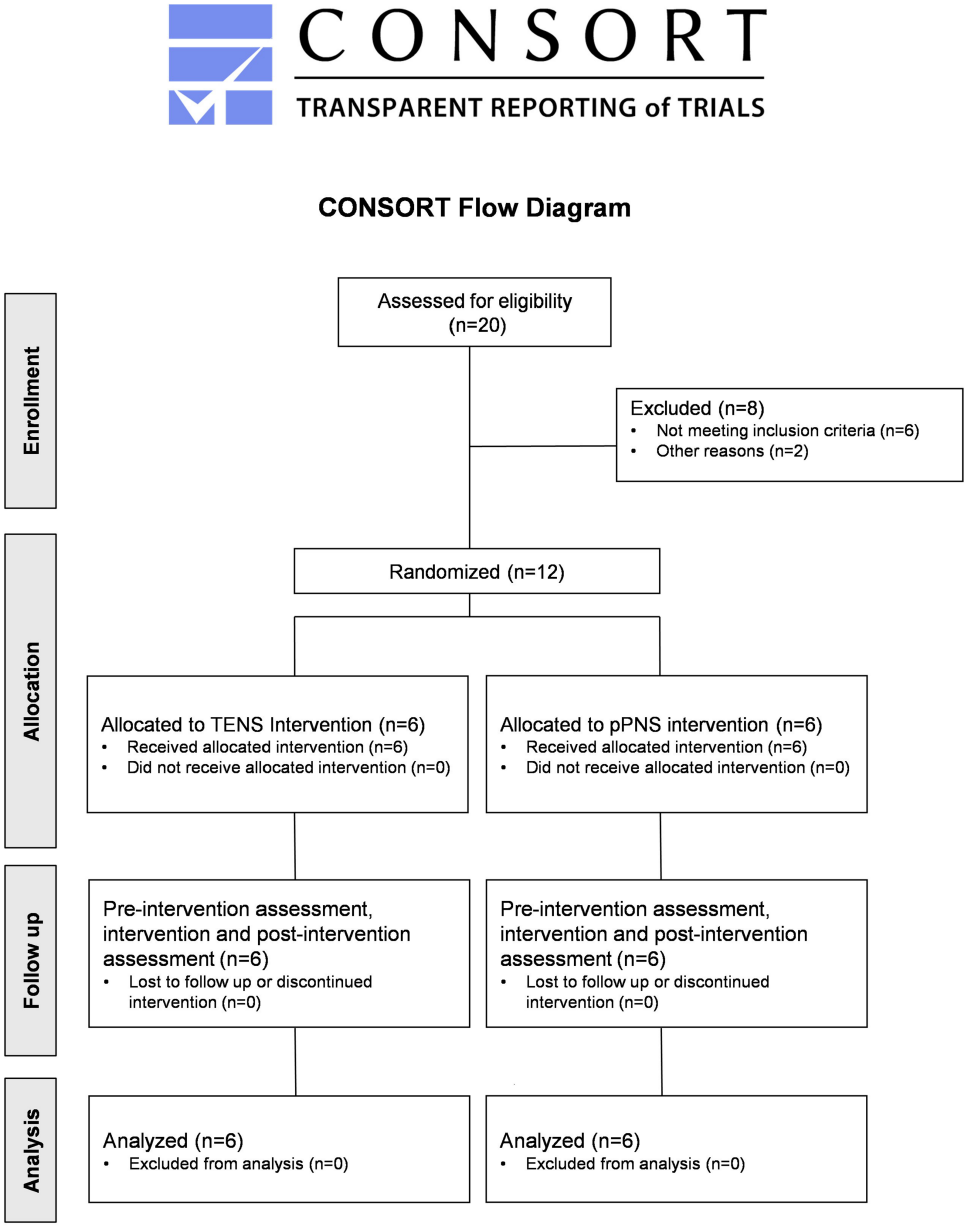
CONSORT flow diagram. Subjects included in the study were randomly allocated to one of two groups: (1) the experimental group, which received ultrasound-guided percutaneous peripheral stimulation, or (2) the comparative control group, which was treated with transcutaneous electrical nerve stimulation.

A randomized parallel-group design was employed for this pilot study, which aimed primarily at evaluating the feasibility of the procedure and refining the design for a future randomized clinical trial (Figure 1). Key considerations for pilot studies and CONSORT guidelines for parallel-group trials were adhered to Moore et al. (46) and Schulz et al. (47). Volunteers participating in the study were subjected to one of the two distinct interventions, with pre- and post-treatment assessments conducted to evaluate the effects on gluteal muscle function. The flow diagram in Figure 1 illustrates the study’s design and the flow of participants.

### Participants and eligibility criteria

The recruitment of participants was conducted in Barcelona, specifically at the Serrahima Athletics Club. Among the initially identified 20 potential candidates, only 12 met the eligibility criteria set forth for inclusion in the study. We had delineated inclusion criteria for recruitment, stipulating that candidates must be (1) adults aged 18–64 years old, (2) amateur athletes, (3) experiencing chronic knee pain, (4) possessing a prior diagnosis related to their knee condition, (5) competent in the execution of the hip thrust exercise, and (6) willing to partake in the intervention protocols (pPNS and TENS).

The exclusion criteria were set to exclude individuals who (1) were underage (less than 18 years old) or elderly (more than 65 years old), (2) presented significant co-existing medical conditions and/or comorbidities, (3) were professional athletes, (4) led a sedentary lifestyle, (5) lacked familiarity with the hip thrust exercise, (6) had needle phobia or rejected peripheral stimulation techniques, or (7) had other considerable contraindications such as a history of knee surgery, current pregnancy, or issues related to blood clotting.

Each participant who met the inclusion criteria and was enrolled in the study provided their informed consent, underwent a preliminary baseline evaluation, and was provided with both a verbal and written comprehensive overview of the study details, including aspects pertaining to data protection and privacy. The demographic and pertinent characteristics of the participants are documented in Table 1.

**Table 1:** Baseline characteristics of study participants.

Descriptive data	pPNS arm	TENS arm	Total
Participants (*n*, %)	6 (50%)	6 (50%)	12
Female (*n*, %)	3 (50% of the group)	4 (66.67% of the group)	7 (58.33%)
Age (years, SD)	25 ± 2.61	23.83 ± 1.46	24.36 ± 2.11
BMI (kg/m^2^)	24.15 ± 0.85	26.25 ± 0.80	25.27 ± 1.27
Knee pain duration (months, SD)	6 ± 3.05	9.17 ± 4.06	7.58 ± 3.90
Knee pain related diagnosis (*n*, %)	Patellofemoral pain syndrome (1; 16.67%)	Patellofemoral pain syndrome (3; 50%)	Patellofemoral pain syndrome (4; 33.33%)
	ITB syndrome (1; 16.67%)	ITB syndrome (1; 16.67%)	ITB syndrome (2; 16.67%)
	Patellar tendinopathy (4; 66.67%)	Patellar tendinopathy (1; 16.67%)	Patellar tendinopathy (5; 41.67%)
		Patellar condropathy (1; 16.67%)	Patellar condropathy (1; 8.33%)
Sports discipline (*n*, %)	Middle distance, 800 m (1; 16.67%)	Middle distance, 800 m (1; 16.67%)	Middle distance, 800 m (2; 16.67%)
	100-m dash (1; 16.67%)	100-m dash (3; 50%)	100-m dash (4; 33.33%)
	Long jump (2; 33.33%)	Triple jump (1; 16.67%)	Triple jump (3; 25%)
	Triple jump (2; 33.33%)	Long jump (1; 16.67%)	Long jump (3; 25%)
Ethnicity	Caucasian (*n* = 6, 100%)	Caucasian (*n* = 6, 100%)	Caucasian (*n* = 12, 100%)

BMI, Body mass index; ITB, Illiotibial band; pPNS, Percutaneous peripheral nerve stimulation; SD, Standard deviation; and TENS, Transcutaneous electrical nerve stimulation.

### Sample recruitment, allocation, and blinding

Given the nature of this pilot study with an emphasis on the feasibility of the methodology, the sample was recruited through a non-probabilistic convenience approach, specifically considering individuals with chronic knee pain. After obtaining informed consent, participants were randomly allocated to either a transcutaneous stimulation intervention or an ultrasound-guided percutaneous peripheral nerve stimulation. To achieve this, a researcher not involved in the assessment and/or intervention carried out concealed allocation using a computer-generated randomized table with GraphPad Software (San Diego, CA, United States), ensuring a balanced 1:1 ratio between the groups. Throughout the study, the clinician responsible for performing the assessments was blinded to both the participants’ conditions and their group assignments. Similarly, a third researcher managed and analyzed the data without knowledge of the group allocations. The only clinician aware of the intervention details was the one executing it, who was not involved in the evaluation or data analysis. The patients undergoing treatment were not informed about the specifics of their assigned intervention to avoid influencing their expectations. However, due to the inherently distinct nature of the invasive vs. transcutaneous interventions and the involvement of peripheral stimulation, achieving effective blinding to the treatment itself proved to be challenging.

### Interventions

**Figure 2:**
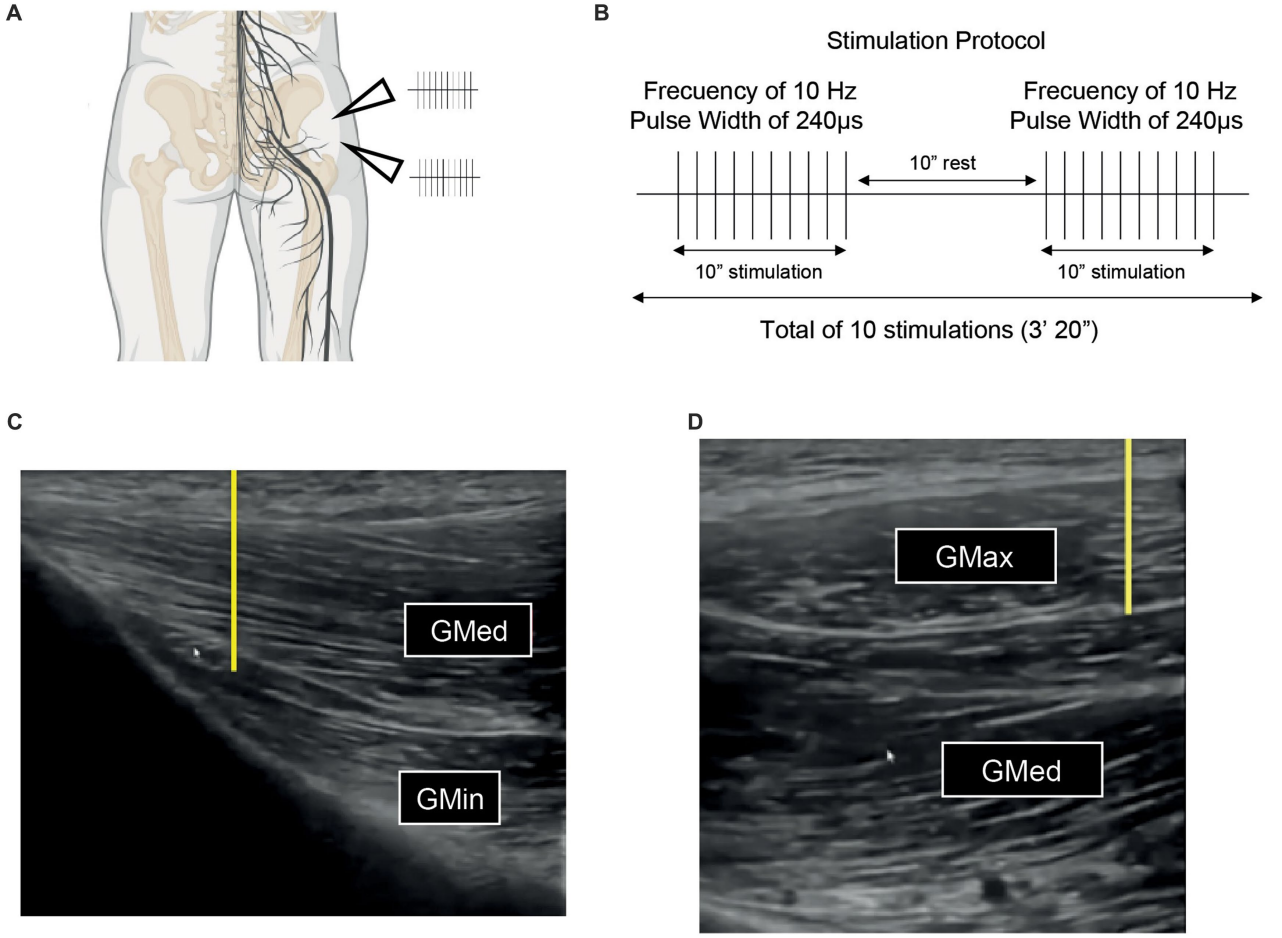
**(A)** Schematic representation of the intervention targeted areas, wherein an ultrasound guided puncture is executed to position the needle proximate to the axons of both the superior and inferior gluteal nerves, aiming for precise percutaneous peripheral nerve stimulation. Superior arrowhead: position of superior gluteal nerve. Inferior arrowhead: position of inferior gluteal nerve. **(B)** Protocol for electrostimulation deployed in each intervention based on the maximal intensity that is bearable at a frequency of 10 Hz and a pulse width of 240 ms, incorporating a series of 10 stimulations lasting 10 s each, interspersed with 10-s intervals of rest. **(C)** Ultrasound visualization of the superior gluteal nerve situated between the gluteus medius and gluteus maximus muscles used to guide the intervention. **(D)** Ultrasound of the inferior gluteal nerve located amidst the muscular layers of the gluteus medius and gluteus minimus, serving to accurately guide the percutaneous peripheral stimulation. Both yellow lines represent the direction of the needle insertion (from proximal to distal). Notably, in both **(C)** and **(D)**, the presence of the vascular package is also discernibly illustrated.

In the experimental cohort, peripheral nerve stimulation was implemented using the portable Esaote MyLabSigma ultrasound system equipped with a linear multifrequency transducer (ranging from 4 to 15 MHz). The procedure commenced with the ultrasound-guided identification of the superior and inferior gluteal nerves (Figures 2A,C,D). Subsequently, the targeted skin area was aseptically prepared using 2% chlorhexidine (LaincoR).

Needle placement was conducted using an in-plane technique with a proximal to distal approach. This involved inserting two needles proximally adjacent to each nerve while ensuring they did not make direct contact with the nerves or the nearby vascular structures. Careful navigation was essential at the intermuscular interface to avoid complications. For the superior gluteal nerve, the connective interface between the gluteus medius and gluteus minimus muscles was precisely localized, while for the inferior gluteal nerve, attention was focused on the area between the gluteus maximus and gluteus medius muscles. The therapist employed an ultrasound-guided approach, integrating specific anatomical knowledge to enhance the accuracy and safety of the procedure (48). Anatomically, the inferior gluteal vessels and the internal pudendal vessels exit the greater sciatic foramen at the inferior level of the piriformis muscle, while the superior gluteal vessels exit the greater sciatic foramen at the superior level, as described by Cocco et al. (49). In each patient, a transverse ultrasound evaluation of the area prior to the procedure was performed to assess the safety of the technique and any anatomical variations. Due to the delicate nature of the gluteal nerves, which are quite thin, it was challenging to discern the characteristic honeycomb pattern. To mitigate the risk of vascular injuries, a color Doppler was utilized prior to the procedure on the targeted area to ensure no blood vessels were present in the vicinity of the needle insertion sites. This precaution helped in preventing vascular accidents during the nerve block administration. Following needle placement, a compensated biphasic asymmetric electrical stimulus, characterized by a positive rectangular phase and a negative triangular phase, was applied. This stimulation was calibrated to a 10 Hz frequency, with a pulse width of 240 μs, and the intensity was finely tuned to the upper limit of the patient’s tolerance threshold (Figure 2B). The objective was to induce the highest possible muscle contraction without eliciting discomfort, aligning with the protocol delineated by Minaya et al. previously (39, 50). The total treatment time consisted of 10 stimulations, each lasting 10 s, with a 10 s rest interval between stimulation (Figure 2B). The delivery of this therapeutic current was facilitated by a certified three-channel electrostimulator (Model ES130) from ITO, employing AGUPUNT needles of dimensions 0.30 mm × 50 mm. This intervention was executed by a clinician with advanced training in ultrasound-guided invasive techniques, ensuring both precision and safety.

In the comparative control group, transcutaneous peripheral stimulation was applied with the same dosage as the experimental group, but without the use of needles. Electrodes were placed over the superior gluteal nerve, specifically halfway between the posterosuperior iliac spine and the ischial tuberosity, and over the inferior gluteal nerve, positioned halfway between the posterosuperior iliac spine and the greater trochanter. The dosage followed the same protocol as the experimental group, with a frequency of 10 Hz, a pulse width of 240 μs, and the intensity set to the maximum level tolerable by the patient to induce painless muscle contractions. For this intervention, standard electrodes from the Rehab Medic Brand were used. For an in-depth visual depiction of the electrode positioning, as well as the placement of needles and/or the probe, please consult Supplementary material. Supplementary Figure 1 provides specific graphical illustrations for understanding the experimental setup and methodology employed in the study.

### Outcomes

**Figure 3:**
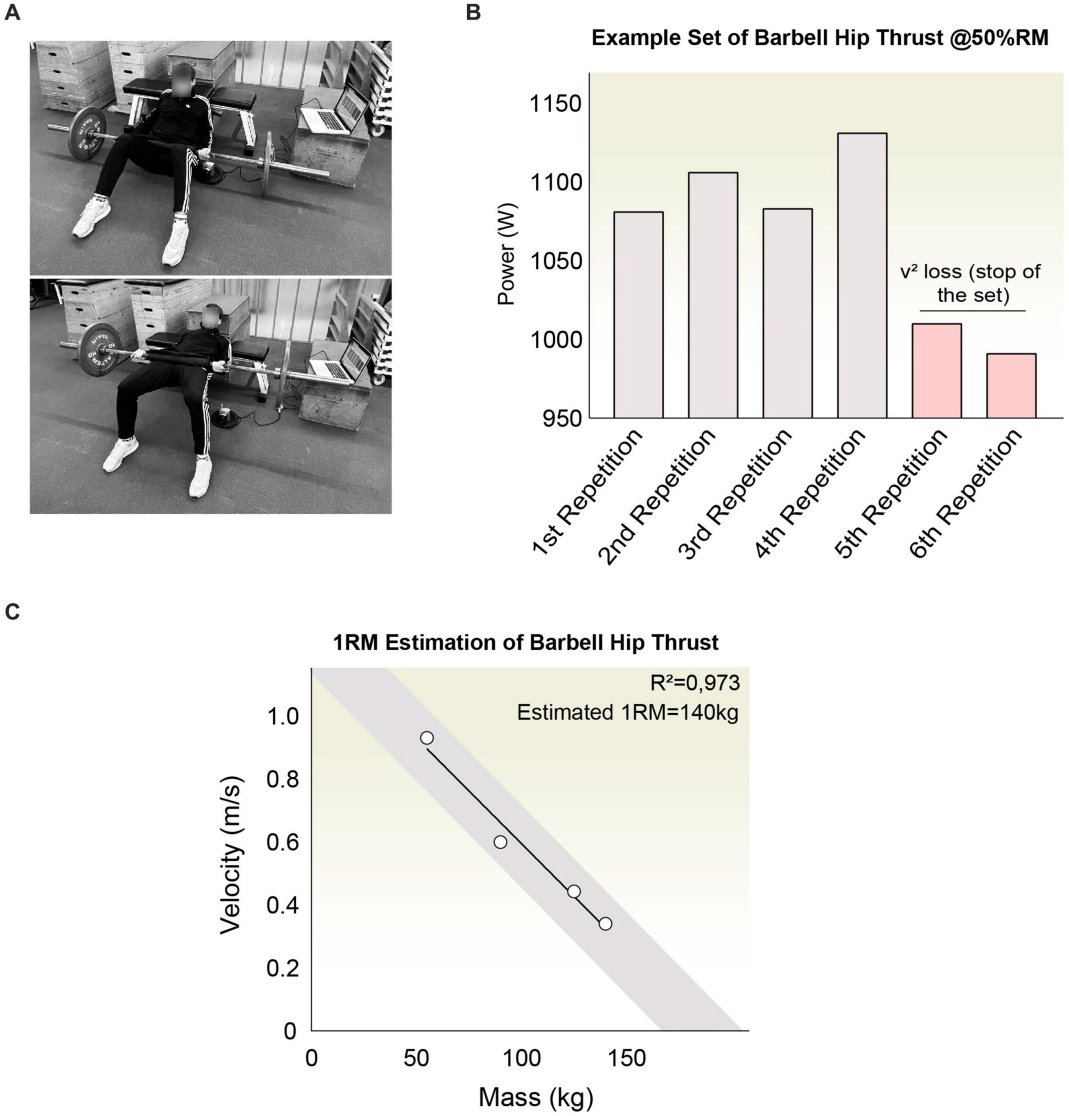
**(A)** Depiction of the task to evaluate the hip extension performance using the Hip Thrust exercise with an Olympic bar. The participant should place the bar equipped with a linear encoder and use a cushioned pad to reduce hip pressure. They should be positioned against a bench with feet set comfortably shoulder-width apart, aiming to execute the hip extension at the highest velocity achievable. **(B)** Outline of a workout set wherein the participant is instructed to generate the utmost power in each repetition. The set concludes once a noticeable decline in speed and performance is detected. The three most effective repetitions from each set are selected for in-depth analysis. **(C)** Depiction of the one-repetition maximum (1RM) estimation process through regression analysis based on the load-velocity curve. A robust *R*^2^ value is attained to ensure the estimation’s accuracy and reliability.

The outcomes evaluated included the velocity of movement execution (m/s), concentric peak power (W), the real-time generated force (N), and the one-repetition maximum (1RM; kg) during hip extension via the execution of a barbell hip thrust at maximal velocity (Figure 3A) (51). The assessments were conducted utilizing a previously validated linear encoder (ChronoJump, Barcelona, Spain) (52, 53). Measurements were captured in real-time with a sampling rate of 1,000 Hz employing the official Chronojump software (version 1.8.1-95), which was designed to compute these metrics. As an open-source platform, Chronojump offers a comprehensive repository containing all relevant codes and formulas used in the measurement process (54). The analytical computations primarily focused on the bar’s vertical displacement (m) while considering the execution time of the concentric phase (s). By analyzing the concentric velocity (m/s) obtained from the linear encoder’s signal conversion, attached to the bar, and factoring in the mass (kg), the software infers the real-time force (N) and power (W). The 1RM was determined indirectly by analyzing the average execution velocity in relation with the mass, utilizing a validated methodology that relies on the load-velocity profile and subsequent linear regression prediction (Figure 3C) (55).

### Procedure

The evaluation of variables was based on the performance of repeated hip extensions using the hip thrust exercise with an olympic barbell. Subjects were positioned with their upper back supported against a bench, and their feet set at shoulder width, oriented either directly forwards or slightly outward (Figure 3A). To alleviate discomfort and facilitate proper positioning over the subjects’ hips, a pad was added to the bar. Participants were instructed to maintain a neutral alignment of both the spine and pelvis throughout the hip extension maneuver. It was emphasized that the concentric phase of the exercise should be executed with maximal explosiveness, whereas the eccentric phase should be rapid yet controlled. For real-time assessment, a linear encoder was attached to the barbell. A preparatory phase of familiarization was conducted on separate days before evaluating the effects of peripheral stimulation to ensure the standardization of technique and execution at peak velocity. Additionally, during this phase, the one-repetition maximum was determined to adjust the relative percent of 1RM for the day of intervention. This evaluation was performed indirectly by analyzing the average execution velocity, utilizing a validated methodology that relies on the load-velocity profile and subsequent linear regression prediction (Figure 3C) (55). Following a warm-up session consisting of three preparatory sets, subjects were instructed to complete four distinct sets of hip thrust with a gradual increase in weight by approximately 20 kg per set, in line with previous research (56). Each set comprised approximately 4–5 repetitions, taking into account the reduction in velocity and power as criteria to conclude the set (Figure 3B). Rest intervals were set to last between 3 and 4 min.

Subsequently, with a week’s interval, the intervention phase was implemented, featuring two assessments pre- and post-stimulation (either pPNS or TENS). In these assessments, three submaximal loads were determined relative to the 1RM previously established, specifically at 30, 50, and 70%. Upon setting these loads, participants underwent a warm-up comprising three preparatory sets, followed by the execution of three test sets at their utmost velocity. Real-time monitoring of the exerted speed was conducted for each set, with the set concluding upon a significant decrease in speed. The statistical analysis took into account the concentric peak power from the three fastest repetitions. The monitoring and recording of the speed, concentric peak power, and strength were facilitated by the Chronojump software, which is freely accessible. Following the pre-intervention test, subjects were administered the designated treatment. A rest period ranging from 5 to 10 min was established between the test and either the pPNS or TENS treatments. After the completion of the intervention, another rest period of 5–10 min was done before repeating the test, adhering to the previously described protocol. The entire study was conducted in Barcelona (Spain) at the facilities of the Serrahima Athletics Club.

### Sample size and sample justification

We aimed to recruit at least one-third of the estimated final sample size for the randomized clinical trial. The sample size was calculated using G*Power and was performed by power analysis, with peak concentric power serving as the primary outcome. We employed a repeated-measures ANOVA for the calculation, setting a statistical power at 80%, significance level at 0.05, with two measurements, two groups, and estimating an effect size of *f* = 0.453. Due to the lack of previous literature, the effect size was extrapolated from the impact of the pPNS intervention on vertical jump power as observed in an earlier pilot study (*n*^2^*p* = 0.170) (38). This provided an initial sample size estimate of 32 patients, leading us to set a recruitment target of at least 12 participants (more than 33%). Given that the estimated sample size may be an underestimation because of the effect size applied, one aim of this pilot study was to gather specific preliminary data to refine the sample size calculation for the upcoming randomized controlled trial (RCT) using both the primary outcome and the targeted sample.

### Randomized pilot trial objectives

Following the extended CONSORT guidelines (44), we clearly state that this pilot study has several aims not centered on directly evaluating the treatment’s effectiveness through hypothesis testing due to the small sample size, which limits statistical power. Therefore, the main goals are to (1) test the feasibility of the methodology and the study protocol, (2) check the feasibility for a randomized clinical trial with an adequately sized sample, and (3) refine procedures and collect data for estimating points and confidence intervals related to future RCTs. Despite this, we aimed to perform inferential statistics in exploratory manner to determine potential trends in treatment effects, being conscious that (1) this is not the main goal of a pilot study and (2) there is a lack of statistical power that compromise external validity.

### Statistical analysis and data visualization

Firstly, the distribution was analyzed through data visualization using Q-Q plots and density plots, along with measures of kurtosis and skewness. Additionally, the normality of the residuals was tested using the Shapiro–Wilk test. Descriptively, the mean, median, mode, and standard deviation for the obtained quantitative measures were analyzed and reflected, and the homogeneity of these variables was examined. In an exploratory manner, inferential statistics were applied to estimate confidence intervals and analyze trends regarding pre- and post-treatment differences, both within and between subjects. Given the non-normal distribution of the data and the pretest-posttest control group design, the non-parametric Mann–Whitney U test was used for between-group comparisons. The Wilcoxon test for related measurements was employed. Data analysis and visualization were conducted using SPSS 23.0 software (SPSS Inc., IBM Chicago, IL, United States) and/or GraphPad Software (San Diego, CA, United States). A 95% confidence interval and an alpha level of 0.05 were assumed for data analysis. Subsequent figure refinement was performed using Adobe Illustrator (San José, CA, United States).

## Results

### Sample demographics and recruitment feasibility

A cohort of 12 individuals suffering from chronic knee pain was recruited, presenting a mean age of 24.36 ± 2.11 years, with females constituting 58, 33% of the sample (Table 1). The diagnose attributed to the pain was pre-established by clinical physicians and subsequently corroborated by the study’s investigators. The distribution of diagnoses encompassed patellar condropathy (8, 33%), patellofemoral pain syndrome (33.33%), patellar tendinopathy (41, 67%), and iliotibial band syndrome (16, 67%). In alignment with the study’s inclusion parameters, participants were active athletes spanning various disciplines: triple jump (25%), long jump (25%), 100 m sprints (33, 3%), and 800 m middle distance running (16, 67%), with all participants identifying as Caucasian. Considering that the present study is a pilot and exploratory research, we observed that approximately 60% of the identified candidates satisfied the inclusion criteria for the inclusion in the study. From a qualitative standpoint, the study’s collaborators deem the sample recruitment process to be pragmatically viable for the forthcoming randomized clinical trial’s execution.

### An exploratory comparison of percutaneous peripheral nerve stimulation and TENS on hip thrust power output

**Figure 4:**
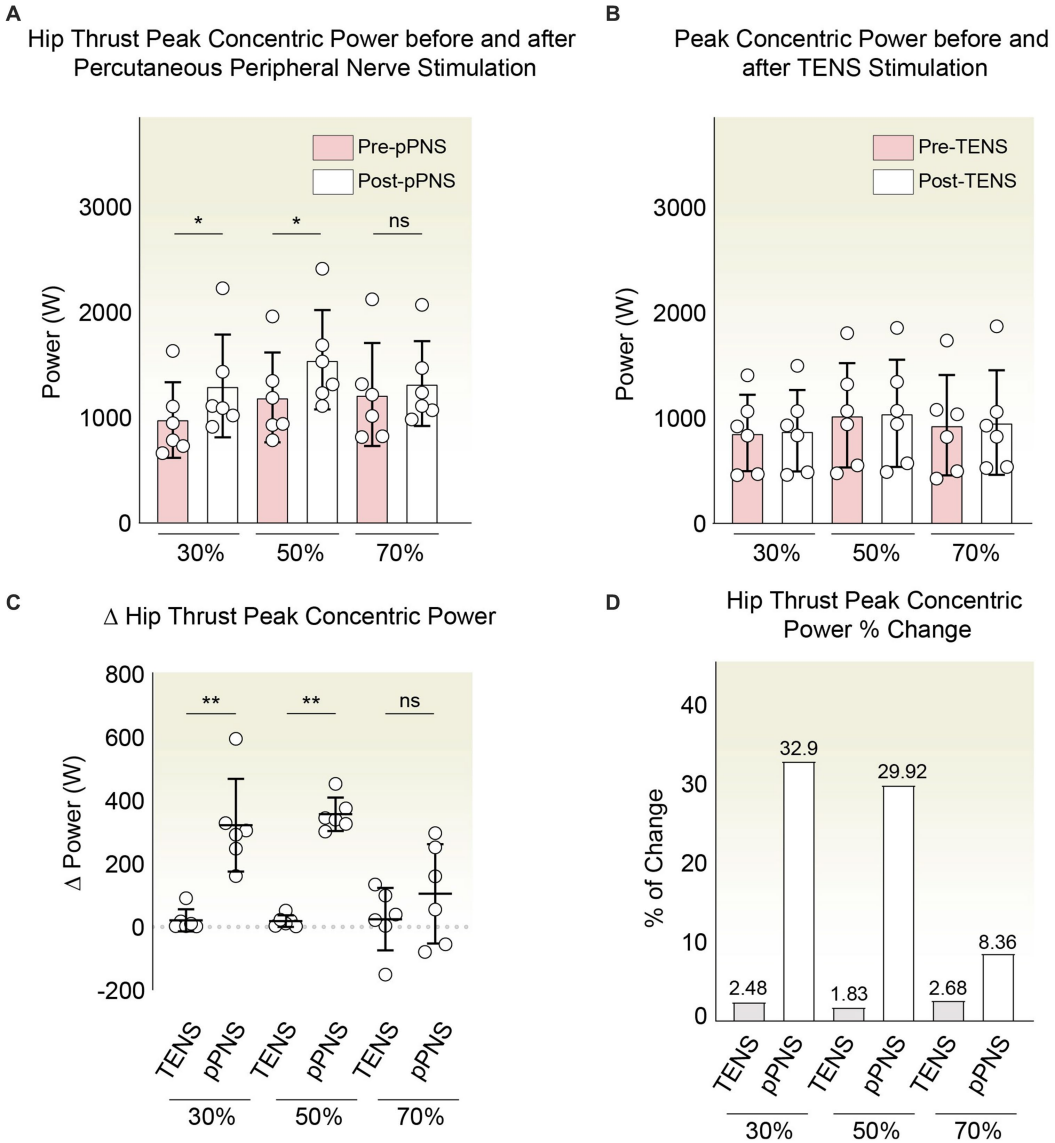
**(A)** Peak concentric power changes post-pPNS. Wilcoxon tests highlighted significant power increases at 30 and 50% of 1RM. No significant changes were noted at 70%. **(B)** Power comparison before and after TENS treatment, showing no significant changes across all tested loads. **(C)** Direct comparison between pPNS and TENS effects on power changes at 30 and 50% of 1RM, using Mann–Whitney U tests to demonstrate significant differences favoring pPNS. **(D)** Normalized percentage improvements in peak power, illustrating more pronounced enhancements with pPNS compared to TENS at 30 and 50% of 1RM. Differences at 70% 1RM were not significant.

In an exploratory manner, we assessed the impact of percutaneous peripheral nerve stimulation vs. TENS on the real-time dynamics of concentric peak power throughout the performance of barbell hip thrust. Following a separate familiarization session, relative load evaluations at 30, 50, and 70% of the 1RM were conducted prior to intervention. Thereafter, targeted stimulation of the lower and upper gluteal nerves was executed via ultrasound-guided pPNS or transcutaneous approach, with a subsequent reevaluation of hip extension performance to discern post-intervention modifications. Preliminary observations revealed distinct efficacy between the interventions. Whereas TENS yielded no significant alterations in pre- vs. post-treatment metrics across all evaluations (Figure 4B), pPNS notably enhanced the concentric phase peak power at 30% (W[+W, −W] = 0[0,21], *p* = 0.031250, *r* = 0.87, 95%CI [−595.4, −161.5]) and 50% of 1RM (W[+W, −W] = 0[0,21], *p* = 0.031250, *r* = 0.8793, 95%CI [−453, −302.23]) (Figure 4A). These outcomes align with the limited preliminary evidence available (38–40). Acknowledging the methodological considerations inherent to pre- and post-comparisons, we further contrasted the generated power differences with those observed in the TENS-treated cohort. We specifically noted a significantly greater alteration in power due to pPNS treatment at the relative percentages of 30% (U = 0, *p* = 0.002165, *r* = 0.81, 95% CI [−504.40, −158.50]) and 50% (U = 0, *p* = 0.002165, *r* = 0.81, 95% CI [−543.10, −159.10]) (Figure 4C). Furthermore, upon calculating the percentage of normalized improvement, pPNS was seen to induce improvements of 32, 9% and 29, 92% at 30 and 50% of 1RM, respectively, as opposed to the 2, 48% and 1, 83% enhancements triggered by TENS (Figure 4D). However, at the 70% 1RM comparison, there were no notable differences in power output change between the groups (U = 12, *p* = 0.3939, *r* = 0.25, 95% CI [−257.30, 101.00]).

### Preliminary assessment of the short-term effectiveness of percutaneous peripheral nerve stimulation vs. TENS in improving hip thrust 1RM strength

**Figure 5:**
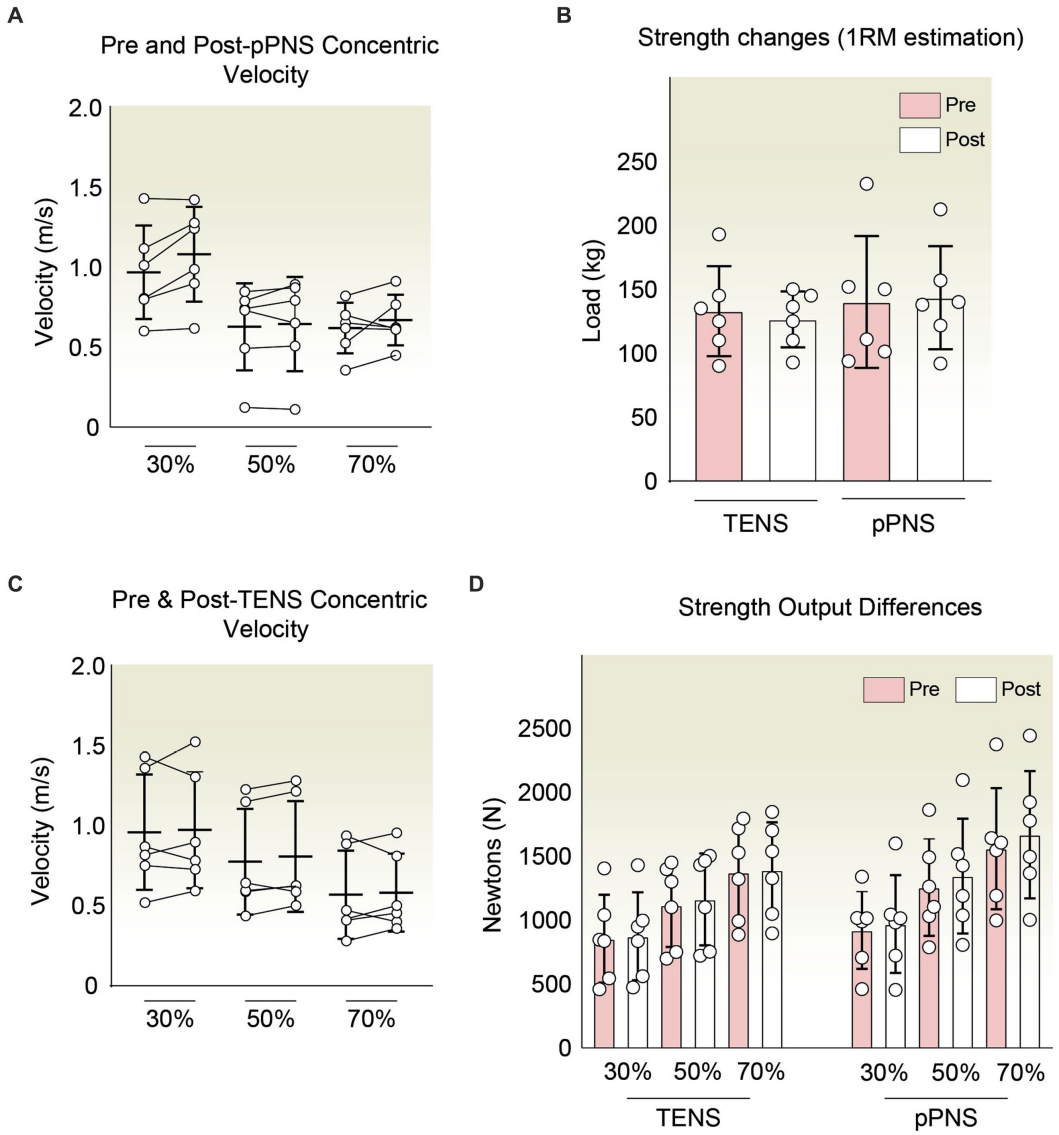
**(A)** Average concentric phase velocity in the hip thrust pre- and post-pPNS application at varying percentages of 1RM (30, 50, and 70%). No significant changes were detected across any condition. **(B)** 1RM estimation comparison using regression analysis on the load-velocity profile before and after pPNS and TENS interventions. **(C)** Mean concentric velocity in the hip thrust before and after TENS application at different relative percentages of 1RM: 30, 50, and 70%. No significant variations were noted in any scenario. **(D)** Force output graphical summary for both groups pre- and post-intervention, indicating negligible differences post-exposure.

Additionally, in light of previous literature regarding electrostimulation and improvements in isometric strength in various contexts (39, 42), we preliminarily characterized whether the use of a 10-s, 10 Hz stimulation protocol via percutaneous stimulation is capable of eliciting an increase in variables associated with strength. To this end, we first assessed whether the average execution speeds across the series were altered by the intervention. In the TENS exposed group, we observed negligible differences between pre and post in meters *per se*cond (m/s): 0.96 ± 0.36–0.96 ± 0.36 (30%RM); 0.78 ± 0.33–0.81 ± 0.35 (50%); 0.57 ± 0.28–0.58 ± 0.24 (70%). Meanwhile, in the pPNS group, we noted a non-significant increase (m/s): 0.97 ± 0.29–1.08 ± 0.30 (30%); 0.63 ± 0.27–0.65 ± 0.30 (50%); and 0.62 ± 0.16–0.67 ± 0.16 (70%). This would imply percent changes in the speed of the concentric phase ranging from 0 to 3.85% in the TENS group and between 3.17 and 11.34% in the pPNS group (Figures 5A,C).

Subsequently, utilizing the aforementioned speed within the context of the load-velocity relationship, we estimated the 1RM for the hip thrust task through linear regression pre and post-intervention using the three evaluated points (30, 50, and 70%) to ascertain if there were trends in the increase of short-term strength. In this regard, we are aware of how several methods, such as proper warm-up, post-activation potentiation (PAP), among others, can enhance short-term performance (57–59). In our case, with the use of TENS, the estimated RM shifted from 133 ± 35.19 to 126.5 ± 21.91 kg. Conversely, with pPNS, it moved from 140 ± 51.56 to 143.53 ± 40.34 kg. However, these were preliminary non-significant changes of −4.80% with TENS and + 2.52% with pPNS (Figure 5B). Similarly, we corroborated the minimal effect on strength with the secondary quantification of strength output in Newtons, obtained indirectly from the weight used and the acceleration applied. Herein, we observed negligible changes in both groups.

Specifically, within the group subjected to TENS, the percentage changes were 2.23% (30%RM), 4.08% (50%RM), and 1.42% (70%RM). On the other hand, we found changes of 5.26% (30%), 7.07% (50%), and 6.95% (70%1RM) in the group treated with ultrasound-guided pPNS. Nonetheless, these preliminary differences are not significantly relevant (Figure 5D).

### Pilot study evaluation: safety, risk and viability considerations

In this pilot study, we assessed aspects concerning the feasibility, safety, and risk factors related to the methodology employed. Our observations revealed that no subjects included in the study experienced any severe or significant complications (Table 2). However, inherent to the nature of the interventions, all participants (*n* = 12) exhibited involuntary muscle contractions during their exposure to the treatments. A minor percentage of the participants (8.33%) reported that these contractions were painful, yet this issue was promptly addressed by adjusting the stimulation intensity, thereby preventing any further complications. Moreover, additional effects such as tingling (16, 67%) and muscle spasms (8.33%) were reported. These, too, were effectively managed by fine-tuning the intensity of the applied stimulation.

**Table 2:** Summary of the adverse effects resulting from the interventions throughout the study.

	pPNS arm	TENS arm	Total
N° of patients w/Serious adverse events	0	0	0
N° of minor events	9	7	16
Innocuous muscle contractions (*n*, %)	6 (100%)	6 (100%)	12 (100%)
Painful muscle contractions (*n*, %)	1 (16.6%)	0 (0%)	1 (8.33%)
Tingling (*n*, %)	1 (16.6%)	1 (16.6%)	2 (16.67%)
Muscle spasms (*n*, %)	1 (16.6%)	0 (0%)	1 (8.33%)

pPNS, Percutaneous peripheral nerve stimulation; SD, Standard deviation; TENS, Transcutaneous electrical nerve stimulation.

These minor events were present only in the pPNS group. Furthermore, the risks associated with the puncture procedure were minimized through the use of ultrasound guidance, which helped in avoiding any unintended contact. The feasibility of the procedure proved to be successful, with no subsequent issues arising. It was crucial, however, to ensure that the subjects achieved the maximum possible speed during the performance assessment task. Failure to do so would invalidate the data collection for the procedure.

## Discussion

To the best of our knowledge, this is the first study aimed at determining the effects of ultrasound-guided percutaneous peripheral nerve stimulation at the level of the gluteal nerves on hip extension performance in individuals with knee pain. In this regard, pPNS has previously been studied for pain relief, showing beneficial outcomes in contexts such as knee osteoarthritis, lower back pain, or elbow pain among others (25, 26, 60–62), capable of alleviating post-operative pain and reducing opioid consumption (25, 61). According to a review, Transcutaneous Electrical Nerve Stimulation (TENS) has demonstrated analgesic effects along with beneficial effects on joint function, quadriceps strength, physical performance, and quality of life in patients with knee osteoarthritis (63). However, our study focused on less investigated variables that could aid functionality and performance in pain contexts (21). It is made clear that this study does not focus on strength improvement as a means to enhance patients’ self-perceived pain relief per se, given the existing controversy regarding the causal relationship between pain improvement by strength enhancement (64, 65). Yet, the inverse relationship seems to be more evident, where we observe that pain can lead to strength loss, reduce the speed and range of movement, and alter the motoneuron discharge rate (66–69), even at a distance from the affected area (70). Nonetheless, we found exceptions, variability, and controversy that warrant attention (3).

Within this context, as an exploratory pilot study, the goal was to preliminarily investigate the trends and changes resulting from the use of pPNS, being aware of the low statistical power that renders the study with no external validity. Indeed, the priority was to establish the feasibility of the trial and to have preliminary data for a better study design in terms of sample size calculation, among others, as critically promoted for pilot studies (46). Despite this, we observed interesting changes where the intervention with pPNS resulted in an increase in concentric power of hip extension at loads of 30 and 50% (Figure 4), improvements that were comparatively greater than those with TENS. Only one study has investigated similar variables, where pPNS applied to the femoral nerve was able to show certain improvements in the countermovement jump (38). A relevant aspect to highlight is that we did not observe this improvement at 70% of 1RM loads.

Interpretations could be premature given the small sample size, and it could be intrinsic to the mechanisms of the intervention and/or attributable to the force-velocity profiles of the athletes themselves, as they all come from disciplines where power prevails over load training (Table 1). We did not evaluate *f*-*v* profiles, but it is known that there are optimal *f*-*v* profiles where athletes with strength deficits could benefit from optimizing such profile with the improvement of force capabilities, and athletes with velocity deficits could take advantage of improving velocity capabilities (71). An aspect that could be somewhat relevant when considering the differences generated by pPNS at different % relative to 1RM.

The efficacy of pPNS in relation to power remains underexplored, yet in the domain of strength we encounter preliminary evidence suggesting the necessity of more rigorous studies (39–43). Typically, strength has been assessed isometrically via dynamometry in most research; however, our approach diverged by deducing it through the athletes’ load-velocity relationship (55). Consequently, we indirectly estimated strength by calculating the one-repetition maximum. Notably, the 1RM measurements before and after the interventions exhibited no significant alterations (Figure 5). Furthermore, by leveraging the acceleration and the mass in kilograms mobilized, we also computed the force output in Newtons, which remained essentially unchanged post-intervention. Thus, our initial findings suggest an inclination toward enhanced power at submaximal loads, albeit without notable shifts in peak strength. It is important to underscore that the corpus of evidence surrounding pPNS does not uniformly report positive impacts on these metrics. For instance, Beltrán et al. (72) noted that low-frequency protocols administered to the median nerve led to diminished grip strength, in contrast to high-frequency applications, which manifested no such effects. Conversely, studies with diminished external validity have reported that pPNS, when applied in pathological contexts, might augment grip strength (43). In patients suffering from lateral epicondylalgia, pPNS targeting the radial nerve was shown to enhance grip strength on the afflicted side, though without significant intergroup disparities (60). Our preliminary data should be viewed as supplementary rather than contradictory, given that the limited existing literature has predominantly focused on maximal isometric strength rather than submaximal load performance. We posit that the observed heterogeneity could potentially be explained by a variety of factors, including (1) inter-regional differences based on the stimulation site and/or the muscles involved, (2) the unique differential effects elicited by varied intervention modalities, (3) the specific demands of the task, or (4) the characteristics of the population subjected to the intervention, among other considerations that may not have been fully accounted for.

Regarding the first point, in accordance with the literature, it is reasonable to consider that the activation of motor units via pPNS entails an altered sequence that deviates from the normal pattern of voluntary activation (73). In this context, the fast motor units (MUs), characteristic of musculature with a higher proportion of Type II fibers, appear to be more susceptible to electrical stimulation, requiring lower intensity for activation (73, 74). Furthermore, the predisposition toward potentiation is a phenomenon traditionally associated with Type II fibers or mixed musculature due to the lower calcium sensitivity and higher levels of myosin light chain kinase in Type II fibers compared to Type I (75). Consequently, fibers more prone to enhancements in calcium sensitivity might be modulating a potentiation effect that allows for improved performance with submaximal loads (75, 76), a phenomenon not only demonstrated by isometric contractions but also through electrical induction (77). However, these potentiation effects should not be mistaken for an actual increase in performance minutes or hours later, as the effects of classical Post-Activation Potentiation are transient, with a notably brief duration (75). Therefore, any hypothetical performance improvement attributed to pPNS would likely be mediated by factors beyond those classically described (75). Nonetheless, it would be plausible to consider that the effectiveness of pPNS on strength variables could be optimized by considering the characteristics of the targeted tissue. In this matter, the proportion of Type II fibers could be key in terms of the potentiation phenomenon, where the gluteal musculature involved in the present study is rather mixed, with post-mortem evaluations showing approximately 52, 4% and 47, 6% of Type I and Type II fibers, respectively (78).

In the second point, we can observe variable effects contingent upon the chosen protocol. In the study presented by Beltrán et al., a protocol designed around pain thresholds, employing low-frequency stimulation with biphasic, symmetric pulses of 250 μs, resulted in diminished strength. However, these decrements in strength were not observed with a high-frequency intervention, which consisted of five bursts at 100 Hz lasting 5 s each, with 55-s intervals in between, calibrated to sensory thresholds that do not elicit pain (72). Indeed, in the traditional conception of TENS, interventions were broadly classified into high frequency, low frequency, or burst-based, which could have more characterized differential effects regarding analgesia (79). Within this context, Johnson et al. outlined the differential impact on peak force and the cumulative force output resulting from five varied protocols of transcutaneous peripheral stimulation (12 pulses at 100, 31, 14, and 5 Hz, and 6 pulses at 14 Hz) (80). In the latter, they observed that the application of 5 Hz tended to generate 50% less potentiation, but once past the 14 Hz threshold, the level of potentiation was similar, where the authors emphasize the importance of the actual number of pulses. In our case, we rely on previous studies by Minaya et al. using 10 stimulations of 10″ with a pulse of 240 μs at a frequency of 10 Hz, where they observed an improvement in isometric strength in the previously affected but not currently painful knee extension (39) (Figure 2). The same protocol was also used in the improvements in vertical jump observed in soccer players (38). Nevertheless, both the site of application and the type of intervention can lead to different scenarios, and the reality is that in the context of ultrasound-guided pPNS, we do not only lack knowledge on the differential effects of the protocols used but also on the dose–response relationship that is typically characterized in the field of pharmacology. Indeed, more knowledge about the latter could optimize the effects of the interventions studied today despite the inter-regional variability that such a relationship could present.

Finally, a simple yet significant aspect must be underscored: the enhancements in strength associated with peripheral nerve stimulation in individuals experiencing pain may be mediated by the modulation of the unpleasant perception of pain and/or the nociceptive neurotransmission itself. In this regard, it would not be surprising that certain interventions, which do not improve strength and/or power in healthy subjects, could do so in individuals suffering from pain. On one hand, we understand that nociceptive information can exert a modulatory effect that alters and redistributes the activity of motor units where there does not necessarily have to be a uniform inhibition *per se* (81). Despite such uniformity, the final output may reflect a net reduction in the capacity to exert force (82). On the other hand, pain as a complex experience entails affective-motivational aspects capable of affecting more complex constructs of the individual (83, 84). In the effort to alleviate nociceptive pain, several studies have demonstrated how techniques such as not only electrical stimulation but also botulinum toxin injections lead to a reduction in symptoms along with an improvement in joint function (85). Neurogenic inflammatory mediators are abundant in the sensory nerve endings of osteoarthritic knees and can sensitize peripheral nociceptors, thereby generating increased nociceptive activation (86, 87). Growing evidence suggests that chronic peripheral nociceptive stimuli play a significant role in triggering both peripheral and central sensitization (88), leading to the onset of neural damage and intrinsic neuropathic pain (89, 90). In turn, the generation of maximum power and strength depends on the motivational state of the subject, including their context. Indeed, a multitude of external and internal variables can the modulate performance in the test (91). Therefore, it is plausible to think that those individuals suffering from chronic pain may not have an adequate pretext (83). In this regard, some authors who have observed greater motor recruitment during knee extension with TENS in surgical contexts attribute it to the reduction of pain itself (37). However, others observe both aspects in a dissociated manner. Therefore, these assumptions are not dichotomous in themselves, emphasizing that all the hypotheses discussed are not exclusive.

In summary, in this pilot study, we demonstrate trends that suggest that pPNS, as a cost-effective intervention applied to the gluteal nerves, could improve power with submaximal loads in hip extension tasks in subjects with chronic knee pain. It would be necessary to investigate whether such improvements have transferability and generalization toward more global and dynamic tasks. Nonetheless, the evidence regarding the application of pPNS in this field is limited, and the mechanisms of action through which we have observed such improvements remain undetermined, with several non-exclusive hypotheses based on previous studies that must be considered.

## Conclusion

This pilot study suggests that ultrasound-guided percutaneous peripheral nerve stimulation could significantly enhance hip extension power at submaximal loads in individuals with chronic knee pain showing superior outcomes compared to Transcutaneous Electrical Nerve Stimulation. Notably, significant enhancements in concentric power were observed at 30 and 50% of one-repetition maximum, although no significant changes were detected in strength assessments, including 1RM estimates, post-intervention. These findings underscore the potential of pPNS to selectively improve muscular performance at reduced loads, suggesting its applicability in targeted therapeutic regimens. Continued research is essential to further establish the robustness of these results and to fully assess the long-term clinical benefits and safety of pPNS.

## Limitations

It must be acknowledged that (1) this constitutes a preliminary study characterized by a limited sample size with a lack of external validity; (2) there is an absence of an additional control group subjected to a placebo pPNS intervention, wherein ultrasound guided needling of the gluteal nerves would be conducted; (3) given the nature of the intervention, it was not possible for participants to remain blinded to the treatment administered (whether percutaneous or transcutaneous); and (4) the particular characteristics of the sample necessitate that participants be adequately acquainted with the task, ensuring their ability to execute hip extension at the maximal velocity achievable, thereby facilitating the acquisition of data of a sufficiently validity.

## Data availability statement

The original contributions presented in the study are included in the article/Supplementary material; further inquiries can be directed to the corresponding author/s.

## Ethics statement

The studies involving humans were approved by Blanquerna School of Health Sciences Research Ethics Committee 450 (Barcelona, Spain). The studies were conducted in accordance with the local legislation and institutional requirements. The participants provided their written informed consent to participate in this study.

## Author contributions

FS: Conceptualization, Writing – original draft, Writing – review & editing. PF: Resources, Writing – review & editing. LM-G: Supervision, Writing – review & editing. GC: Data curation, Formal analysis, Visualization, Writing – original draft. JP: Data curation, Formal analysis, Investigation, Visualization, Writing – original draft, Writing – review & editing. AP-D: Funding acquisition, Investigation, Methodology, Project administration, Supervision, Validation, Writing – review & editing.

## Glossary

AMI: Arthrogenic muscle inhibition
BMI: Body mass index
CER: Research ethics committee
CONSORT: Consolidated standards of reporting trials
GABA: Gamma-aminobutyric acid
ITB: Iliotibial band
kg: Kilograms
m/s: Meters per second
MU: Motor unit
N: Newtons
PAP: Post-activation potentiation
pPNS: Percutaneous peripheral nerve stimulation
RCT: Randomized controlled trial
RFD: Rate of force development
SD: Standard deviation
SPIRIT: Standard protocol items: recommendations for interventional trials
TENS: Transcutaneous electrical nerve stimulation
W: Watts
1RM: One-repetition maximum
