# Supplementary material for: Ultrasound-guided gluteal nerves electrical stimulation to enhance strength and power in individuals with chronic knee pain: a randomized controlled pilot trial
Source: Front Med (Lausanne). 2024 Jul 3;11:1410495. doi: 10.3389/fmed.2024.1410495 (PMC11251890; doi:10.3389/fmed.2024.1410495)
Supplement: Supplementary file 1 [file Image_1.pdf]

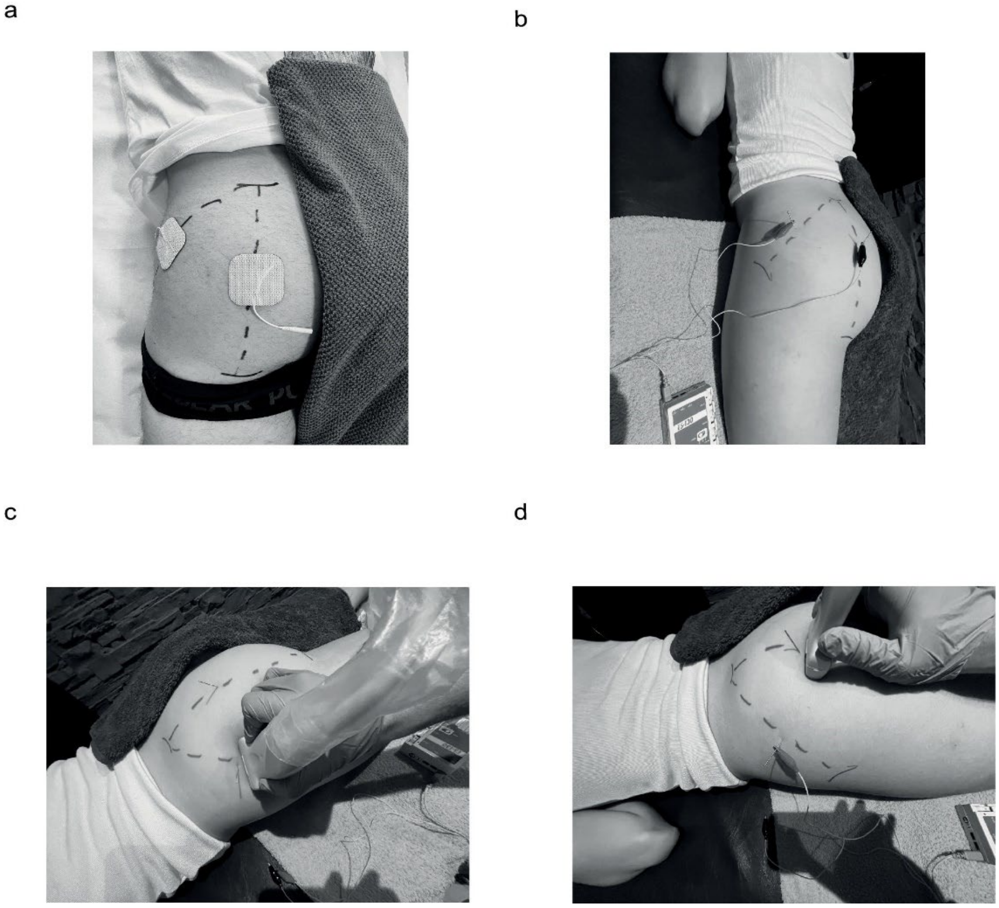

Supplementary Figure 1. (A) Schematic depiction of electrode placement for the TENS group intervention. (B) Schematic depiction of needle placement for the pPNS intervention. (C) In-plane approach for accessing the superior gluteal nerve. (D) In-plane approach for accessing the inferior gluteal nerve.
